# Neuroimaging Features of Immune Reconstitution Inflammatory Syndrome in a Patient with AIDS Successfully Treated for Neurocryptococcosis

**DOI:** 10.1155/2013/970141

**Published:** 2013-09-01

**Authors:** Antônio O. F. da Silva, Luciano Z. Goldani

**Affiliations:** Section of Infectious Diseases, Hospital de Clínicas de Porto Alegre, Universidade Federal do Rio Grande do Sul, Ramiro Barcelos 2350, 90035903 Porto Alegre, RS, Brazil

## Abstract

The use of highly active antiretroviral therapy (HAART) has significantly reduced the incidence and progression of HIV-associated cryptococcosis. However, an early complication of HAART is the immune reconstitution inflammatory syndrome (IRIS), which may affect the CNS. The authors report a patient successfully treated for cryptococcosis and HIV who presented a late manifestation of IRIS. Neuroimaging aspects and management of CNS-IRIS in this patient are discussed in this paper.

## 1. Introduction

Cryptococcosis is a global invasive mycosis associated with significant morbidity and mortality [1]. The use of highly active antiretroviral therapy has significantly reduced the incidence and progression of HIV-associated cryptococcosis [2]. However, an early complication of ART is the immune reconstitution inflammatory syndrome (IRIS), which may affect the central nervous system (CNS). The authors report a patient successfully treated for cryptococcosis and HIV who presented a late manifestation of IRIS. Neuroimaging aspects and management of CNS-IRIS in this patient are also discussed in this paper.

## 2. Case Report

A 26-year-old HIV-positive female was brought in to the emergency department with a history of tonic-clonic seizures 2 days earlier and bilateral lower limb weakness. Although the patient was alert and oriented, she was unable to walk. The patient had been diagnosed HIV-positive three months earlier and started taking antiretroviral medications since then, when she was admitted to hospital with a history of fever and headache. Her lymphocyte CD4+ count was then 57 cells/mm³, and HIV viral load was 89,290 copies/mL. Serum cryptococcal latex antigen test (CLAT) was positive (titer 1 : 10,000). Lumbar puncture (LP) was performed (opening pressure 780 mm H_2_O), and fungal culture was positive for *Cryptococcus neoformans*. She was treated with serial LP, amphotericin B plus 5′flucytosine for 14 days and then started on oral fluconazole 800 mg/day. CSF cultures were negative for *C. neoformans* after 14 days of amphotericin B and 5′flucytosine treatment. She was discharged for followup at the outpatient clinic and started on HAART (zidovudine, lamivudine, and efavirenz).

**Figure 1:**
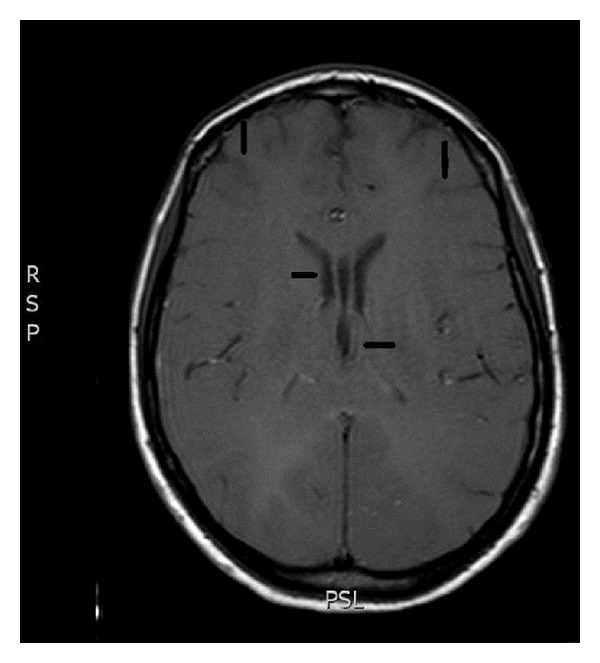
Brain MRI (T1-weighted image) showed diffuse swelling of the brain due to extensive edema of the white matter characterized *loss* of gray-white junction and collapse of the ventricles (arrows).

**Figure 2:**
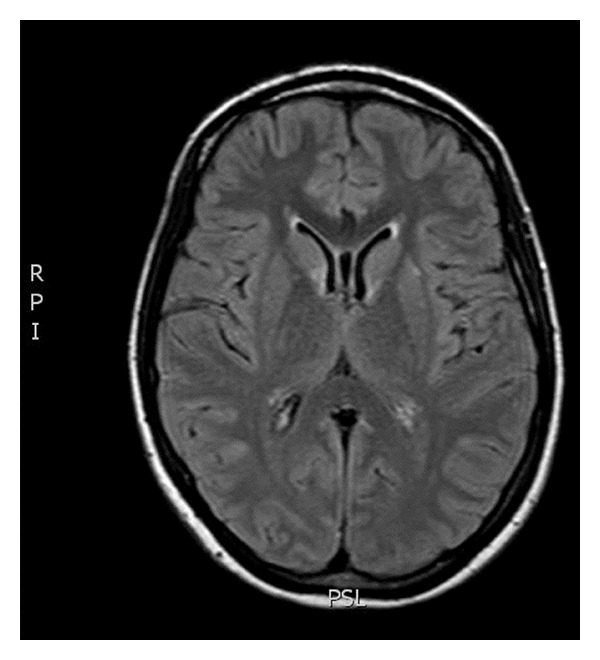
Brain MRI (T1-weighted image) was repeated and showed marked decrease in the volume of cerebral edema following 10 days of therapy with prednisone. Note the increased space of the gray-white matter junction and the ventricles (arrows).

At the present admission, LP was again performed and opening pressure was 260 mm H_2_O, and CSF was negative for fungi, viruses, bacteria, and mycobacteria. The patient was taking oral fluconazole 800 mg/day and HAART for two months. Serum CLAT was still positive at a titer lower than 1 : 10. Brain MRI showed diffuse swelling of the brain due to extensive edema of the white matter and collapse of the ventricles (Figure 1). She was started preemptively on amphotericin B plus 5′flucytosine but had no neurological improvement. Lymphocyte CD4+ count was 192 cells/mm^3^ and HIV viral load was undetectable. A diagnosis of immune reconstitution inflammatory syndrome (IRIS) was made and prednisone (80 mg/day) started. The patient recovered fully from her neurological symptoms after 10 days of treatment with prednisone. Brain MRI was repeated two weeks after admission and showed marked decrease in the volume of cerebral edema (Figure 2). Patient was discharged for follow-up taking oral fluconazole 800 mg/day and HAART.

## 3. Discussion

Cryptococcal immune reconstitution inflammatory syndrome (IRIS) may present as a clinical deterioration or new presentation of cryptococcal disease following initiation of HAART and is believed to be caused by recovery of cryptococcus-specific immune responses [3]. The time of onset of IRIS after HAART varies widely, from 4 days up to 3 years, with a median time ranging from 1 to 10 months [4]. Reported CD4 counts prior to ART are typically below 50 cells/mm^3^, but pre-ART viral loads (VL), virologic outcomes, and follow-up CD4 counts are inconsistently reported. In case reports, individual data are usually reported, giving a median baseline CD4 count of 28 cells/mm^3^ and VL of 5.6 log_10_ copies/mL, and CD4 count at event of 162 cells/mm^3^ and VL of 2.4 log_10_ copies/mL. While recognition of CNS-IRIS may be difficult in patients with a recent history of cryptococcosis taking antifungal agents and HAART, the onset of new or progressive clinical symptoms, the appearance of cerebral interstitial edema, the onset of new lesions, mass effect, and restricted diffusion in infections not typically characterized by these findings should raise the suspicion of cryptococcal CNS-IRIS [5]. Cryptococcal IRIS becomes a clinical diagnosis and there is no specific laboratory test for detection and diagnosis. The most important recommendations for cryptococcal IRIS management are clinical identification and prevention. When it is causing severe disease such as increased intracranial pressure, cryptococcal CNS-IRIS management requires serial lumbar puncture and the use of an anti-inflammatory strategy with corticosteroids (prednisone or dexamethasone) to prevent further host damage while on treatment with highly effective antifungal therapy including amphotericin B and 5′flucytosine [6]. It has been demonstrated, even in HIV-infected individuals, that corticosteroids can be safely administered for limited durations as long as viral load is controlled. Except in severe cases, HAART therapy should not be interrupted in patients with cryptococcal CNS-IRIS. Specific treatment for minor IRIS manifestations is not necessary because they will resolve spontaneously in days to weeks.

Physicians living in areas with high incidence of cryptococcosis and HIV with increasing use of highly active antiretroviral therapy should be able to recognize the clinical and neuroimaging features and select the appropriate management of cryptococcal CNS-associated IRIS.
